# The Ring-Necked Pheasant (*Phasianus colchicus*) Industry within the United Kingdom and the Threat Posed by *Mycoplasma gallisepticum*: A Review

**DOI:** 10.3390/vetsci9080391

**Published:** 2022-07-29

**Authors:** Matthew J. Balfour

**Affiliations:** St David’s Poultry Team Limited, Exmouth EX8 5AN, UK; matthew.balfour@stdavids-poultryteam.co.uk

**Keywords:** *Mycoplasma gallisepticum*, ring-necked pheasants, infectious sinusitis, review, epidemiology, clinical signs, diagnosis, control

## Abstract

**Simple Summary:**

In ring-necked pheasants (*Phasianus colchicus*), *Mycoplasma gallisepticum* (MG) infection is frequently associated with infectious sinusitis. This condition causes swelling of the infraorbital sinuses, upper respiratory distress, depression and variable levels of mortality, and is considered one of the most important clinical and economic diseases of pheasants. This review provides a brief overview of the structure of the UK pheasant industry, with reference to the various stages within the supply chain, common diseases and challenges facing the industry. The current understanding of MG transmission, prevalence, clinical expression, diagnosis and control strategies in pheasants is subsequently summarised. In addition, this review aims to assess the current gaps in knowledge relating specifically to MG in pheasants, with reference and extrapolation where appropriate to data gathered from other species. This review will be of particular interest to clinicians in the field when planning MG control or treatment strategies in pheasants. It may also be of academic interest as it provides a summary of poorly studied areas.

**Abstract:**

In ring-necked pheasants (*Phasianus colchicus*), *Mycoplasma gallisepticum* (MG) infection is frequently associated with infectious sinusitis. This condition causes swelling of the infraorbital sinuses, upper respiratory distress, depression and variable levels of mortality, and is considered one of the most important clinical and economic diseases of pheasants. This review provides a brief overview of the structure of the UK pheasant industry, with reference to the various stages within the supply chain, common diseases and challenges facing the industry. The current understanding of MG transmission, prevalence, clinical expression, diagnosis and control strategies in pheasants is subsequently summarised. In addition, this review aims to assess the current gaps in knowledge relating specifically to MG in pheasants, with reference and extrapolation where appropriate to data gathered from other species.

## 1. Introduction

*Mycoplasma gallisepticum* (MG) causes significant economic losses within the UK poultry industry and within poultry industries globally. The bacterium is unusual in its complete lack of a cell wall, its ability to cause a persistent infection within a host and its potential for both horizontal and vertical transmission [1]. It has been known to infect a wide range of avian species and is associated with a range of clinical or subclinical presentations. In broiler chickens it is associated with chronic respiratory disease (CRD), reduced weight gain and carcass condemnations [2] whilst in layers it may have a substantial negative impact on egg production [3]. In turkeys MG infection is often associated with respiratory disease [4]. Another feature of MG is its propensity combine with other pathogens, for example *Escherichia coli* or Avian Metapneumovirus, to cause more severe disease [2,5].

In ring-necked pheasants (*Phasianus colchicus*), infectious sinusitis, frequently associated with MG infection [6], is one of the most important pheasant diseases in terms of its adverse impact on health and welfare, its economic cost to the industry and its contribution to increased antibiotic usage within the industry. The first published description of infectious sinusitis infection in UK pheasants occurred in 1958 [7], although the author noted anecdotal evidence of the disease from industry sources over many preceding years. Further work published in 1961 suggested a link with MG when the bacterium was cultured from birds showing clinical signs [8]. Since then, various studies have strengthened the association between MG and infectious sinusitis [6,9] and have further explored the transmission, prevalence, clinical expression, diagnosis and control strategies in pheasants. This paper gives an overview of the historical and current knowledge summarised in these papers, as well as an assessment of future considerations regarding MG infection in pheasants. In addition, the paper summarises the current structure of the pheasant industry within the UK with reference to the various stages within the supply chain, common diseases and challenges facing the industry.

## 2. Structure of UK Pheasant Industry

The UK pheasant industry is a sizable entity which is responsible for the annual release of approximately 41 million captive-bred pheasants into the wild. It is a key component of the overall UK shooting industry which contributes an estimated £2.5 billion to the UK economy every year [10]. Compared to many other livestock industries the UK pheasant industry suffers high levels of disease and mortality, due to myriad factors which include: very few licensed medications, a complete absence of any pheasant-licensed vaccines, relative lack of high-health status breeding stock and a lack of enforced industry standards for management and biosecurity. As well as the negative implications for bird health, welfare and economics, this widespread prevalence of disease results in high antibiotic usage. In 2018, the total quantity of antibiotics administered to gamebirds (pheasants and partridges) within the UK was 9.7 tonnes [11]—more than three times higher than the antibiotic usage in the entire UK chicken layer industry over the same period. This is of concern to human health due to the potential development of antimicrobial resistance (AMR) [11].

The pheasant industry in the UK is broadly divided into pheasant laying farms, hatcheries, rearing farms and shoots. Many enterprises are integrated with two of more of these activities on-site. Within laying farms, breeding birds are housed in either single-harem outdoor wire cages, usually containing nine individuals [12] or multi-harem grass runs, containing up to 100 individuals, for the purpose of obtaining fertile eggs for hatching. Adult pheasants in these systems are either caught-up from the wild between December and March or maintained in permanently captive flocks [13]. Feed usually consists of a commercial pelleted layer pheasant ration. Eggs are laid for eight to 12 weeks between April and June [14], after which the laying hens are either sold to shoots or kept in captivity until the next breeding season. A mortality rate of 5% is considered acceptable in a grass run system that houses caught-up pheasants for 5 months [13]. Common causes of mortality within both cage and grass run systems are infectious sinusitis, nephritis, *E. coli* septicaemia, helminth infection, reproductive tract disorders and trauma [13].

Hatcheries obtain eggs from laying farms and incubate them for 25 days. Day-old chicks are then transported to rearing farms in temperature-controlled vehicles. During the hatching process it is essential that high standards of biosecurity and hygiene are maintained so as to prevent cross contamination and chick disease. Common diseases of chicks which can be linked to poor hygiene and/or management during incubation and hatching include yolk sac infection, non-starters and rotavirus [15].

When day-old chicks reach rearing farms, they are placed in brooding sheds. These sheds vary greatly in design and may house several hundred to several thousand birds. Heat is commonly supplied by a gas brooder and bedding may be comprised of wood chips/shavings, straw or chopped cardboard. Usually there is a grass run attached which birds are able to access from around three weeks of age onwards, depending on the weather [15]. On many sites, at around three weeks of age, small plastic bits are routinely fitted into the beak in a process known as ‘bitting’. These prevent the beak from closing completely and have the effect of reducing feather pecking behaviour. Bits are removed from the beak immediately prior to release [16]. Feed is initially a commercial game chick crumb which is then transitioned to a commercial grower pellet. During the rearing phase pheasants are very susceptible to disease and mortality can reach high levels. The main diseases of concern are enteritis, coccidiosis, spironucleosis, infectious sinusitis and helminth infection [15].

At six to eight weeks of age the pheasant poults are transferred to outdoor release pens on a shooting estate. These pens comprise fenced areas of land, varying enormously in size, but usually consisting of a mixture of woodland (with a mixture of tree/shrub species), an area of cover crop and an area of open ground. Covered feeders and drinkers are placed strategically within the release pens and are topped up as required. During this phase, feed is initially provided as a commercial pelleted ration which is usually transitioned to wheat after several weeks following placement. After two to four weeks in these pens the pheasants are released into the wild. Within these pens diseases of primary concern are coccidiosis, spironucleosis, infectious sinusitis and helminth infection [15].

## 3. Epidemiology

### 3.1. Association with Infectious Sinusitis

MG is considered within the veterinary community to be the primary cause of the upper respiratory condition known in pheasants as infectious sinusitis [9,17]. Studies have repeatedly shown an association between infectious sinusitis incidents and diagnosis of MG (*p* < 0.05) [9]; ranging from 59% of the 22 incidents tested by PCR in one study [6] to 50% of 28 incidents tested by PCR or RSAT in another [9].

However, other respiratory pathogens are frequently involved. These include avian metapneumovirus (*p* < 0.01) as diagnosed by presence of antibody titers, avian coronavirus (*p* < 0.05) as diagnosed by reverse transcriptase-polymerase chain reaction (RT-PCR) and Pasteurella (*p* < 0.05) as diagnosed by culture from the sinus [9].

### 3.2. Transmission Routes

To a certain extent transmission routes in pheasants must be extrapolated from the plentiful work carried out on chickens. The experience of veterinarians in the field suggests that these routes appear similar, however peer reviewed data from pheasants is scant and extrapolation from chicken studies must be treated with a degree of caution.

For the field clinician, MG control is made particularly challenging by its potential for both vertical and horizontal transmission, as well as the existence of a permanent carrier state post-infection [18,19,20]. MG was first shown to be capable of vertical transmission by work published in 1954 which referred to a ‘pleuropneumonia-like organism’ (later re-named MG) capable of causing CRD in chickens [20]. The following year data was published which demonstrated routes of horizontal transmission; namely indirect airborne transmission and direct contact with infected chickens [19,21].

Subsequent work has highlighted the importance of fomites in the indirect horizontal spread of MG within the chicken industry, for example stockpersons moving between populations, movement of machinery and prompt re-stocking of previously contaminated sheds. An important factor relating to this route of transmission is survivability outside of the host. Generally, this is short: MG cannot be re-isolated from straw, cotton or rubber after 2 days, from human hair after 3 days or from feathers after 4 days [22]. However, there are examples where MG has been found viable for longer periods of time, such as in chicken faces where it has isolated after 7 days at room temperature and in egg material where it has been isolated for up to 7 weeks [23]. Clearly some fomites, in particular egg material, are at a heightened risk for transmitting disease.

Within the UK pheasant industry, transmission vertically from an infected hen to the chick is prevalent and is a frequent cause of infectious sinusitis outbreaks on the rearing field. The rate of vertical transmission observed in clinical practice correlates with that described in chickens: a peak of 50% in untreated acutely infected females, falling to 3% within 2–3 months following infection [17]. Following vertical infection of a cohort of chicks on a rearing field, horizontal spread by direct or indirect routes is likely to occur, leading to a far larger outbreak. Clinical signs may appear suddenly, in particular if there has been a concurrent stressor (e.g., bitting) or co-infection with another respiratory pathogen [17].

Pheasants which are not exposed to MG through vertical transmission or on the rearing field remain at risk of infection by horizontal transmission during the release phase on a shoot. This may come in the form of direct or indirect contact with infected wild pheasants or other wild bird species, which may or may not display clinical signs. Within the UK, carrion crows (*Corvus corone*), rooks (*Corvus frugilegus*) and jackdaws (*Corvus monedula*) have all been shown to carry MG [24,25] and therefore form a potential reservoir of disease. Furthermore, experimental infection of red-legged partridges (*Alectoris rufa*), which are frequently reared and released alongside pheasants, has been shown to produce clinical signs and a carrier state similar to that seen in pheasants [26].

Worldwide, MG has been found in 56 (mostly wild) avian species from 1951 to 2018 by culture, PCR, serum plate agglutination test (SPA), haemagglutination inhibition test (HI), enzyme-linked immunosorbent assay (ELISA), growth inhibition test (GI) or tube agglutination assay (TA) [27]. For example, in Belgium it has been detected in wood pigeon (*Columba palumbus*), mallard duck (*Anas plathyrhynchos*), Eurasian magpie (*Pica pica*), grey heron (*Ardea cinerea*) by ELISA [28] whilst within the USA and Canada, MG is now widespread within wild house sparrow (*Carpodacus mexicanus*) and goldfinch (*Carduelis tristis*) populations where it is associated with conjunctivitis [29]. Based on findings from other countries it is plausible that a larger MG surveillance survey of the wild UK bird population may expand the list of known carrier species.

The transmission of MG from pheasants to commercial free-ranging poultry is frequently cited as a potential source of infection [1]; however, there is also the chance of horizontal transmission in the other direction. This was demonstrated in the USA where MG of poultry origin has been shown to infect house finches with subsequent genomic evolution of the bacterium [30].

### 3.3. Prevalence in the Wild and Captive UK Pheasant Population

Although MG is considered a major disease of pheasants within the UK, published data describing its true prevalence within wild and captive populations is scant.

In one study, which assessed caught-up laying pheasant populations from 1995–1997 on the same estate in south west Scotland, infectious sinusitis was determined to be (despite antibiotic treatment) the leading infectious cause of mortality and culling in adult laying pheasants. The disease accounted for a cumulative mortality 0.1% to 0.4% depending on the sampling year [13]. Other studies have tested birds from multiple sources already showing clinical signs of infectious sinusitis and recorded a prevalence of 50 to 59% by PCR or RSAT [6,9]. Of course, the true prevalence across clinically unaffected birds will be substantially lower and it is likely there are both seasonal and regional variations in prevalence.

## 4. Clinical Expression in Pheasants

In pheasant chicks infected at day old, clinical signs may be noticed initially at 10 days post-infection [31]. This compares with a 14-day period between infection and clinical signs appearing in work carried out on older 8- to 10-week-old birds [32].

**Figure 1:**
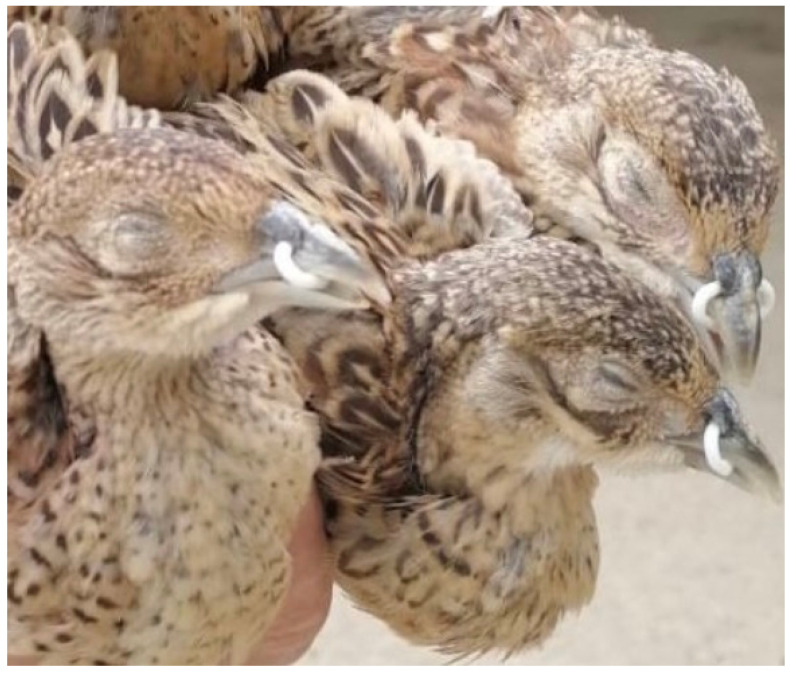
Pheasants aged 6 weeks with clinicals signs of MG infection.

Clinical expression of infectious sinusitis is often seen following periods of stress. Typically, in adult pheasants it is seen within the week following catching up from the wild or at the onset of lay [13,33]. In the initial stages, disease is characterised by an accumulation of clear mucus in the nasal cavity and infraorbital sinuses with concurrent conjunctivitis and epiphora. As time goes on the contents of the sinuses become caseous, sinus swelling develops and tracheal lesions may become apparent [15,32]. Birds become progressively depressed, show signs of upper respiratory distress and lose weight [13]. An example of 6-week-old pheasants in the initial stages of disease expression is illustrated in Figure 1.

Morbidity in a flock may be high but mortality is variable [13] and may depend on the presence/absence of co-infections or other stressors [9]. There is also the possibility that different MG strains display different levels of pathogenicity in pheasants as they do, for example, in turkeys [34].

## 5. Diagnosis in Pheasants

Various diagnostic tests may be utilised for the diagnosis of MG infection in pheasants, however not all will be appropriate in every scenario.

Culture remains the gold standard for detection of viable MG within a host. The culture process generally involves swabbing the infraorbital sinuses, eyes and trachea from whole head submissions, then plating the sample onto mycoplasma agar and incubating at 37C in a carbon dioxide incubator [6]. The process may take several weeks and is a necessity if antibiotic minimum inhibitory concentration (MIC) testing or autogenous vaccine production is required. In clinical cases of infectious sinusitis 27% of incidents were diagnosed as MG positive using culture alone, however when PCR was introduced for subsequent cases, this figure rose to 59%. Therefore, there is some evidence that culture may be less sensitive at diagnosing MG than PCR in clinically affected birds [6]. It should also be noted that antibiotic treatment will significantly reduce the probability of culture success in MG-infected birds [35]

PCR testing for MG in pheasants using commercial kits is a readily available, rapid and relatively inexpensive method of diagnosis. Moreover, it appears to be the test with the highest sensitivity and specificity compared with other diagnostic methods [27]. Sample submission from the field to the testing laboratory is relatively straightforward and can be in the form upper respiratory tract swab samples or whole heads [6]. It should be noted that antibiotic treatment will significantly reduce the probability of a PCR positive result in MG-infected birds [35].

Serological tests available for MG diagnosis include ELISA, HI and SPA. Since seroconversion in pheasants occurs 22 days after challenge [32] their use is not appropriate in the early stages of MG infection. SPA testing is known to produce false-positive results due to its cross-reactivity [36]. For HI and ELISA testing, cut-off titer levels for a positive result in pheasants remains uncertain and may differ between laboratories, leading to variability in reported results [27]. Due to their low cost, serological tests are of particular use when screening a flock of uncertain health status for MG infection, provided a sufficient sample size is used. If extrapolating from the European MG control programme for reproduction stock in chickens, a gold-standard sample size would be 60 birds per epidemiological unit every 90 days. However, due to the fact that the specificity of all serological tests is lower than 100%, false-positive results can be expected when sampling any large number of individuals. These false-positive results can either be negated by further confirmatory testing (PCR or culture) or simply by accepting a low number of positive results before a flock is considered positive—for example OIE suggest up to 10% positive SPA results are acceptable in a negative flock [37,38].

## 6. Control Strategies

### 6.1. Management and Biosecurity

For the pheasant laying sites, rearing sites and shoots the gold standard for MG control is to keep disease out in the first place through stringent biosecurity and good management practices. However, if this is not possible then every effort should be made to reduce horizontal transmission within the site.

Since disease often starts in laying flocks and is transmitted vertically, MG control at this stage of the supply chain is particularly important. An overwintered closed laying flock of known health status is preferred, however the practice of ‘catching-up’ from the wild for breeding purposes is still widespread within the industry [13]. If this is the case, then ideally a single supplier of ‘caught-up’ birds should be used and the MG status of the birds investigated. If more than one shoot is used, then efforts should be made to keep breeding birds from each source apart [39]. Increased levels of stress or disease on the laying farm will increase the likelihood of flare-up and spread from carrier birds, therefore the following should be encouraged: drawing up a Veterinary Health Plan, correct transportation and handling of birds, acceptable stocking densities, a water sanitisation strategy, a vermin control plan and prompt investigation of any respiratory disease outbreak [33,37].

On rearing sites and shoots similar principles apply to those described above. Due to practical considerations, multiple age groups and species from different sources may be present on the same rearing site and, in this scenario, separation should be maintained by housing different groups in different enclosures [39]. On the shoot separation is particularly difficult due to the fact that birds have been released into the wild, and here the placement of birds from only one trusted source is of particular importance.

Across all parts of the supply chain biosecurity is of utmost importance; both to prevent initial introduction of MG and to reduce its spread within a site. As a minimum this should include limited visitor access, provision of site-specific footwear and protective clothing and protective measures against wild bird access to the bird area (particularly where open feeders and drinkers are sited) [39].

### 6.2. Antibiotic Therapy

If birds within a laying or rearing site becomes infected with MG the owner may implement a culling and re-stocking policy in the hope of fully eliminating disease [33]. However, in many cases antibiotic therapy is employed, ideally in conjunction with the management and biosecurity principles described above. The practice of antibiotic therapy is common in other poultry industries affected by MG and has been shown to suppress clinical signs, reduce transmission and reduce economic losses associated with the disease. Unfortunately, this approach cannot eradicate disease from an infected flock and the issue of antimicrobial resistance (AMR) has become increasingly prevalent. In particular, resistance to enrofloxacin, tylosin and oxytetracycline has now developed across a wide range of geographical regions and in a variety of poultry species [40].

The only UK licensed antibiotic for MG in pheasants is “Aivlosin 625 mg/g granules for drinking water for pheasants” which contains tylvalosin and is licensed at 25 mg tylvalosin/kg bodyweight for 3 consecutive days. This product has been shown to have an MIC value of 0.002 µg/mL to 0.008 µg/mL, depending on the strain tested. In treated infected birds it has been shown to significantly reduce clinical signs associated with MG, significantly improve bodyweight gain and significantly reduce isolation by culture and detection by PCR compared with untreated infected birds [35]. Although the effect of tyvalosin on vertical or horizontal transmission has not been studied, it is possible that the reduction in MG detection seen in treated birds may lead to a reduction in transmission rates.

In the past other antibiotic products have been used under the cascade for treatment of MG in pheasants. These include lincomycin and spectinomycin (in combination), tiamulin and enrofloxacin [33]. It should be noted that no form of antibiotic treatment has been known to eliminate the MG carrier state seen in pheasants post-infection.

### 6.3. Vaccination

MG vaccination in chickens with both live and inactivated vaccines is widespread. In the case of inactivated vaccination, it has been shown to produce a blood antibody response, substantially reduce the associated clinical signs [3,41] and substantially reduce vertical MG transmission [3].

Some clinicians have claimed success at reducing the incidence of infectious sinusitis in pheasant flocks following the use of inactivated chicken-licensed commercial or autogenous MG vaccines. However, in the absence of published data their efficacy remains unproven [42]. The use of live chicken-licensed commercial MG vaccines is discouraged by the British Veterinary Poultry Association (BVPA) due to uncertainty regarding their efficacy and possible spread to non-target birds [39].

## 7. Future Considerations

Overall, it can be seen that MG poses a significant ongoing and future threat to the UK pheasant industry. With its potential for both vertical and horizontal transmission, large-scale MG outbreaks can appear suddenly, particularly if a prominent laying site supplying numerous rearing sites has suffered an outbreak. Prompt investigation of respiratory disease outbreaks as well as the establishment of sound biosecurity and management protocols is therefore of vital importance. At present there is no government-sponsored eradication program for MG in pheasants and so the disease must be tackled by co-operation and the establishment of acceptable standards within the industry.

It is clear that there is considerable potential for future research into MG infection in pheasants. Future work of particular importance to the industry would include an evaluation of the significance of different routes of transmission, the effect of antibiotic treatment on vertical and horizontal transmission and the effect of vaccination on the expression of clinical signs and transmission of MG.

Going forwards the author is unaware of any further developments in the licensing of antibiotics or vaccines for MG in pheasants.

## References

[1] Levisohn S., Kleven S.H. (2000). Avian mycoplasmosis (*Mycoplasma gallisepticum*). Rev. Sci. Et Tech. (Int. Off. Epizoot.).

[2] Kleven S.H. (1998). Mycoplasma in the etiology of multifactorial respiratory diseases. Poult. Sci..

[3] Glisson J.R., Kleven S.H. (1984). *Mycoplasma gallisepticum* vaccination: Effects on egg transmission and egg production. Avian Dis..

[4] Jordan F.T. (1975). Immunity to mycoplasma infections of the respiratory system in the domestic fowl and turkey. Dev. Biol. Stand..

[5] Naylor C.J., Al-Ankari A.R., Al-Afaleq A.I., Bradbury J.M., Jones R.C. (1992). Exacerbation of *Mycoplasma gallisepticum* infection in turkeys with rhinotracheitis virus. Avian Pathol..

[6] Bradbury J.M., Yavari C.A., Dare C.M. (2001). Mycoplasmas and respiratory disease in pheasants and partridges. Avian Pathol..

[7] Keymer I.F. (1958). A survey and review of causes of mortality in British birds and the significance of wild birds as disseminators of disease. Vet. Rec..

[8] Keymer I.F. (1961). Infectious sinusitis of pheasants and partridges. Vet. Rec..

[9] Welchman D., Bradbury J., Cavanagh D. (2002). Infectious agents associated with respiratory disease in pheasants. Vet Rec..

[10] (2014). Public and Corporate Economic Consultants, The Value of Shooting. http://www.shootingfacts.co.uk/pdf/The-Value-of-Shooting-2014.pdf.

[11] (2019). Veterinary Medicines Directorate, UK Veterinary Antibiotic Resistance and Sales Surveillance Report. https://assets.publishing.service.gov.uk/government/uploads/system/uploads/attachment_data/file/950126/UK-VARSS_2019_Report__2020-TPaccessible.pdf.

[12] (2012). Department for Environment Food and Rural Affairs, Study to Provide Scientific Evidence on Whether Cage- Based Breeding for Pheasants and Partridges Can Fully Meet Birds’ Needs, and If Not to Identify Best Practice for Improving the Breeding Environment for Gamebirds. http://sciencesearch.defra.gov.uk/Default.aspx?Menu=Menu&Module=More&Location=None&Completed=0&ProjectID=17541.

[13] Pennycott T.W. (2000). Causes of Culling and Mortality in Adult Pheasants. Vet. Rec..

[14] Matheson S., Donbavand J., Sandilands V., Pennycott T., Turner S. (2015). An ethological approach to determining housing requirements of gamebirds in raised laying units. Appl. Anim. Behav. Sci..

[15] (2008). Welchman, Diseases in Young Pheasants. Practice.

[16] Butler D., Davis C. (2010). Effects of plastic bits on the condition and behaviour of captive-reared pheasants. Vet. Rec..

[17] Welchman D., Brzozowska A. (2019). Health issues in breeding gamebirds. Vet Rec..

[18] Fahey J.E., Crawley J.F. (1956). Studies on chronic respiratory disease of chickens. VII. The nature of infection with pleuropneumonia-like organisms. Can. J. Comp. Med. Sci..

[19] Fahey J.E., Crawley J.F. (1955). Studies on chronic respiratory disease of chickens. V. Airborne spread of the CRD agents. Can. J. Comp. Med. Sci..

[20] Fahey J.E., Crawley J.F. (1954). Studies on chronic respiratory diseases of chickens. III. Egg transmission of a pleuropneumonia-like organism. Can. J. Comp. Med. Sci..

[21] Beard C.W., Anderson D.P. (1967). Aerosol Studies with Avian Mycoplasma. I. Survival in the Air. Am. Assoc. Avian Pathol..

[22] Christensen N., Yavari C., McBain A., Bradbury J. (1994). Investigations into the survival of *Mycoplasma gallisepticum*, *Mycoplasma synoviae* and *Mycoplasma iowae* on materials found in the poultry house environment. Avian Pathol..

[23] Chandiramani N.K., van Roekel H., Olesiuk O.M. (1966). Viability Studies with *Mycoplasma gallisepticum* under DIfferent Environmental Conditions. Poult. Sci..

[24] TPennycott W., Dare C.M., Yavari C.A., Bradbury J.M. (2005). *Mycoplasma sturni* and *Mycoplama gallisepticum* in wild birds in Scotland. Vet. Rec..

[25] Bradbury J.M., Dare C.M., Yavari C.A., Forrester A. Evidence of *Mycoplasma gallisepticum* in British Wild Birds. Proceedings of the Abstracts of the 13th International Congress of the International Organisation for Mycoplasmology.

[26] Ganapathy K., Jones R.C., Bradbury J.M. (1998). Pathogenicity of *Mycoplasma gallisepticum* and *Mycoplasma imitans* in red-legged partridge (Alectoris rufa). Avian Pathol..

[27] Sawicka A., Durkalec M., Tomczyk G., Kursa O. (2020). Occurrence of *Mycoplasma gallisepticum* in wild birds: A systematic review and analysis. PLoS ONE.

[28] Michiels T., Welby S., Vanrobaeys M., Quinet C., Lieze R., Lens L., Martel A., Butaye P. (2016). Prevalence of *Mycoplasma gallisepticum* and *Mycoplasma synoviae* in commercial poultry, racing pigeons and wild birds in Belgium. Avian Pathol..

[29] Fischer J.R., Stallknecht D.E., Dhondt A.A., Converse K.A. (1997). Mycoplasmal conjuctivitis in willd songbirds: The spread of a new contagious disease in a mobile host population. Emerg. Infect. Dis..

[30] Delaney N.F., Balenger S., Bonneaud C., Marx C.J., Hill G.E., Ferguson-Noel N., Tsai P., Rodrigo A., Edwards S.V. (2012). Ultrafast Evolution and Loss of CRISPRs Following a Host Shift in a Novel Wildlife Pathogen, *Mycoplasma gallisepticum*. PLoS Genet..

[31] Forrester C.A., Davis C., Bradbury J.M. Pathogenicity Studies on *Mycoplasma gallisepticum* and Avian Pneumovirus in Pheasants. Proceedings of the Abstracts of the 15th International Congress of the International Organization for Mycoplasmology.

[32] Forrester C.A., Davis C., Gough R.E., Bradbury J.M. *Mycoplasma gallisepticum* and Pheasant Coronavirus Act Synergistically to Cause Respiratory Disease in Pheasants. Proceedings of the Abstracts of the 16th International Congress of the International Organization for Mycoplasmology.

[33] Pennycott T.W. (2001). Disease control in adult pheasants. Practice.

[34] Lin M.Y., Kleven S.H. (1982). Pathogenicity of two strains of *Mycoplamsa gallisepticum* in turkeys. Avian Dis..

[35] Forrester A.C., Bradbury J.M., Dare C.M., Domangue R.J., Windsor H., Tasker J.B., Mockett A.P. (2011). *Mycoplasma gallisepticum* in pheasants and the efficacy of tyvalosin to treat the disease. Avian Pathol..

[36] Feberwee A., Mekkes D.R., de Wit J.J., Hartman E.G., Pijpers A. (2005). Comparison of culture, PCR, and different serologic tests for detection of *Mycoplasma gallisepticum* and *Mycoplasma synoviae* infections. Avian Dis..

[37] Feberwee A., de Wit S., Dijkman R. (2022). Clinical expression, epidemiology, and monitoring of *Mycoplasma gallisepticum* and *Mycoplasma synoviae*: An update. Avian Pathol..

[38] OIE (2018). Avian Mycoplasmosis (Mycoplasma gallisepticum and Mycoplasma synoviae).

[39] BVPA (2018). BVPA Working Group Recommendations for Mycoplasma Management in Gamebirds.

[40] Taiyari H., Faiz N.M., Abu J., Zakaria Z. (2021). Antimicrobial minimum inhibitory concentration of *Mycoplasma gallisepticum*: A systematic review. J. Appl. Poult. Res..

[41] Hildebrand D.G., Page D.E., Berg J.R. (1983). *Mycoplasma gallisepticum* (MG)—Laboratory and field studies evaluating the safety and efficacy of an inactivated MG bacterin. Avian Dis..

[42] Welchman D. (2016). Diseases in Gamebirds: An update. Practice.

